# A comprehensive study of speed prediction in transportation system: From vehicle to traffic

**DOI:** 10.1016/j.isci.2022.103909

**Published:** 2022-02-12

**Authors:** Zewei Zhou, Ziru Yang, Yuanjian Zhang, Yanjun Huang, Hong Chen, Zhuoping Yu

**Affiliations:** 1 School of Automotive Studies, Tongji University, Shanghai 201804, China; 2 Department of Aeronautical and Automotive Engineering, Loughborough University, LoughboroughLE11 3TU, UK; 3 College of Electronics and Information Engineering, Tongji University, Shanghai201804, China

**Keywords:** Algorithms, Engineering, Transportation engineering

## Abstract

In the intelligent transportation system (ITS), speed prediction plays a significant role in supporting vehicle routing and traffic guidance. Recently, a considerable amount of research has been devoted to a single-level (e.g., traffic or vehicle) prediction. However, a systematic review of speed prediction in and between different levels is still missing. In this article, existing research is comprehensively analyzed and divided into three levels, i.e. macro traffic, micro vehicles, and meso lane. In addition, this article summarizes the influencing factors and reviews the prediction methods based on how those methods utilize the available information to meet the challenges of the prediction at different levels. This is followed by a summary of evaluation metrics, public datasets, and open-source codes. Finally, future directions in this field are discussed to inspire and guide readers. This article aims to draw a complete picture of speed prediction and promote the development of ITS.

## Highlights

• A comprehensive review is provided for speed prediction in and between different levels
• Existing speed prediction methods at different levels are systematically surveyed
• The future directions of speed prediction in the transportation system are elaborated

## Graphical abstract

**Figure:**
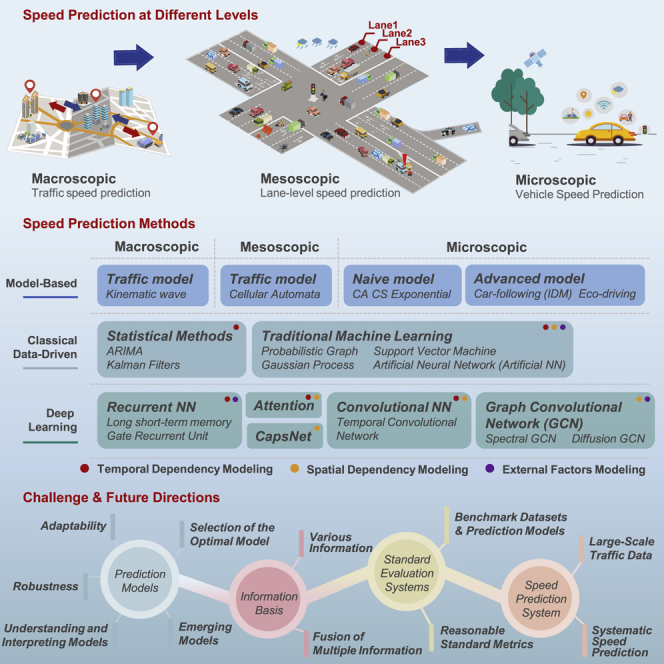

## Introduction

The transportation system is the blood vessel of cities. However, with the rapid gathering of population toward cities, the problems of urban traffic, such as congestion, pollution, and accidents, have significantly affected the traffic efficiency and city development. To address these issues, intelligent transportation system (ITS) has attracted extensive interests (Yuan and Li, 2021), which provides efficient traffic service and management. Different from constructing new infrastructures (e.g., roads and bridges), limited by space and high cost (Lana et al., 2018), ITS can collect and process diverse data through intelligent infrastructure and advanced algorithms to improve traffic efficiency. The prediction of the traffic states online or offline is a fundamental part of ITS (Nagy and Simon, 2018), which enables the current traffic service and management far sight. Among the traffic states, speed is the basic property of dynamic traffic, which reflects vehicle motion and traffic efficiency (Qu et al., 2021). Furthermore, route planning and traffic intervention in advance based on speed prediction is an effective way to improve traffic efficiency, reduce energy consumption, and improve participants' experience.

Speed prediction aims to estimate the speed of traffic participants in a future period based on current and historical traffic states. Meanwhile, prediction can be classified into three categories according to the different scales, i.e., traffic speed prediction (macro), vehicle speed prediction (micro), lane-level speed prediction (meso). Traffic flow consisting of multiple vehicles as the target of traffic-speed prediction describes the dynamic characteristics of traffic in a macroscopic view. Thus, the time horizon of this prediction can range from minutes to hours, even days (Zang et al., 2019), as shown in Table 1. The accurate short-term prediction of traffic speed in minutes can support traffic management, such as the optimization of signal timing and traffic resources allocation (Wang et al., 2016). Moreover, traffic participants can utilize the traffic trend to plan their travel (Park et al., 2014). On the other hand, the traffic patterns captured by long-term prediction in hours help to understand the traffic and support transportation planning (Nagy and Simon, 2018). For example, the prediction methods can capture the main factors causing congestion in a specific scenario and support the planning of new roads to relieve congestion. However, vehicle speed prediction focuses on the identification of the future micro speed patterns. The future information of vehicle speed is useful for trajectory planning and eco-driving (Huang et al., 2018; Ye et al., 2019). Especially for the hybrid electric vehicles, the speed prediction is a key part of energy management strategy (EMS) to improve the powertrain efficiency (Sun et al., 2015a; Liu et al., 2021). Collision risk can be reduced by a reliable speed prediction as well (Liu et al., 2019). The traffic flow of lanes at mesoscale, as the target of lane-level prediction, describes the dynamic traffic in more detaile than macro traffic speed and captures the interaction between lanes, such as lane change. Therefore, it is able to provide the speed information at lane level, which is essential for lane-based traffic applications such as high-precision navigation and intelligent driving (Gu et al., 2019b; Shen et al., 2021). Table 1 shows the detailed comparison of the speed prediction at different levels in terms of the prediction target, speed characteristics, time horizon, and application.

**Table 1:** Comparison of the speed prediction at different levels

	Traffic-speed prediction (Macro)		Lane-level speed prediction (meso)		Vehicle-speed prediction (micro)	
Target	Average Speed of Multiple Vehicles in Road & Network		Average Speed of Multiple Vehicles in Road Lane		Single Vehicle Speed	
Characteristic	External Factors Influence Dynamic Spatio-Temporal Dependency		Interactions between Lanes Lane-level Spatio-Temporal Dependency		Driver and Vehicle Influence Dynamic Temporal Dependency	
Time Horizon	05 min	Yu, 2021 Qu et al., 2021 Zhang et al., 2020a	01-30 s	Lu et al., 2020c	05 s	Shih et al., 2019 Shen et al., 2018b Liu et al., 2018b
	15 min	Wu et al., 2019 Zhao et al., 2020b; Chen et al., 2020a Zheng et al., 2020; Xie et al., 2020	02-10 min	Raza and Zhong, 2017 Lu et al., 2020a, 2020b Tao et al., 2020	15 s	Guo et al., 2019a Hyeon et al., 2019
	30 min	Li et al., 2018b; Ge et al., 2019 Huang et al., 2020 Zhang et al., 2020d	10-20 min	Ke et al., 2020b Lu et al., 2020b	30 s	Sun et al., 2017 Gaikwad et al., 2020 Xie et al., 2017; Park et al., 2011
	60 min	Min and Wynter, 2011 Li et al., 2018b Zheng et al., 2020	30 min	Ke et al., 2018	50 s	Zhou et al., 2019a Lian et al., 2017
Application	Traffic Management(Traffic Resources Allocation and Signal Timing Optimization)	Wang et al., 2016 Yu et al., 2017, 2021 Polson and Sokolov, 2017	High-precision Traffic Management	Lu et al., 2020b, c Raza and Zhong, 2017 Gu et al., 2019b	Eco-Driving(Energy and Thermal Management)	Guo et al., 2019a He et al., 2021 Sun et al., 2015b
	Travel Planning (Efficient and Comfortable)	Liebig et al., 2017 Yu et al., 2019a Yang et al., 2020	Lane-level Travel Planning	Ke et al., 2020a Tao et al., 2020 Lu et al., 2020a Liu and Juang, 2021	Collision Risk Estimation	Vogel, 2003 Polychronopoulos et al., 2007 Barrios and Motai, 2011
	Transportation Planning	Nagy and Simon, 2018 Liao et al., 2018				

Because of the influence of various contextual factors, e.g., weather and accidents, the traffic is a chaotic dynamic system in essence, which brings complex nonlinearity and uncertainty to speed variables. Therefore, how to discover the hidden patterns in traffic is the most critical problem for speed prediction. To address this, plenty of prediction methods have been proposed, and the first category belongs to the theoretical model-based methods. Such deductive methods (Wang and Shi, 2013) are easy to realize but inevitably suffer from inaccuracy because of strong artificial assumptions (Shen et al., 2018a). In addition, those methods are powerless to describe various factors and model their uncertainty. Unlike the model-based methods, the data-driven ones inductively learn the law from available data and capture the traffic pattern without explicit models. Such methods can model various factors gracefully and obtain high-quality results. However, the interpretability and adaptability of such methods are still a puzzle. With the rapid development of data acquisition and computing power, the research priority of speed prediction gradually transforms from analytical methods to data-driven methods (Vlahogianni et al., 2014). Meanwhile, the data-driven methods have evolved from statistical methods to traditional machine learning and recently to deep-learning methods. Because of its powerful capacities to fit non-linear functions and analyze big data, deep-learning methods are considered as the most promising approach (Yin et al., 2021a). In addition, because the characteristics of speed predictions at different levels are not the same, specific methods should meet the different demands of speed prediction in applications.

### Difference from existing surveys on speed prediction

Regarding traffic-speed prediction, reference (Vlahogianni et al., 2014) summarized ten challenges based on the research from 2004 to 2013. However, it could not cover the popular methods based on deep learning. Reference (Yuan and Li, 2021) provided a survey from the data layer to application layer of traffic prediction, and the traffic data format was discussed in detail in (Nagy and Simon, 2018). But the prediction methods are not the emphasis in their discussion. Thus, study (Miglani and Kumar, 2019) presented a survey of deep-learning methods used for traffic prediction in autonomous vehicles, and study (Tedjopurnomo et al., 2020) focused on three common neural network methods. However, those researches paid insufficient attention to recent advances, e.g., attention and graph-based learning models. The deep-learning methods were categorized into five generations to describe the research trend in (Lee et al., 2021), and reference (Ye et al., 2020) provided a survey specifically on graph-based deep learning. Meanwhile, research (Yin et al., 2021a) conducted experiments to compare different deep learning methods, and research (López Manibardo et al., 2021) discussed the pros and cons of deep-learning methods in detail. Most of these works focus on traffic prediction (e.g., flow, speed, and demand prediction), but the detailed review of speed prediction is still absent.

With respect to vehicle speed prediction, parametric and non-parametric methods were compared over 1–10 s horizon in (Lefevre et al., 2014), which indicated that non-parametric methods are suitable for the long-term prediction. Reference (Liu et al., 2019) classified the prediction methods of vehicle speed into two categories: deterministic and stochastic prediction, and reference (Zhou et al., 2019c) provided an overview of driving prediction (e.g., speed, acceleration) and its application. Meanwhile, study (Huang et al., 2017) presented a review of model predictive control based strategies in EMS, and simply reviewed the prediction methods of vehicle-speed. The existing works about vehicle speed prediction focus on the application of EMS, but the systematic discussion of the prediction methods is still missing. Furthermore, there are fewer studies on lane-level speed prediction since it has been enjoying attention in recent years. In conclusion, most existing literature focuses on the speed prediction only at a single level. The community lacks a systematic and comprehensive survey to explore the differences and similarities of speed prediction at different levels. Our research aims to fill this gap and inspire future researchers.

### The contributions of this article

• This article systematically summarizes the speed prediction in the transportation system into three categories according to the scale: Traffic (macro), vehicle (micro), and lane-level (meso). The similarities and differences of speed prediction at different levels are explored to promote a comprehensive understanding.
• Various influencing factors of speed are summarized and a review of different speed predictions is provided based on the type and amount of information utilized. Meanwhile, this article describes the theory, characteristics, and variants of each method to inspire further research.
• The existing evaluation metrics for prediction models are collected, and the public traffic datasets and open-source models are also organized to facilitate future work’s development and experimentation.
• This article concludes with a discussion of the challenges and future directions for the speed prediction problem in transportation.

### Organization of this article

Definitions and preliminaries section presents the definition and characteristic of speed prediction at different levels. The influencing factors of speed are also demonstrated. Besides, Prediction methods of traffic speed section Prediction methods of vehicle speed section, and Lane-level speed prediction section provide an overview of prediction methods at different levels in terms of model-based and data-driven methods. Evaluation section provides evaluation metrics, public datasets, and codes in the literature and discusses the existing challenges and future directions. Finally, Conclusion section draws a conclusion of this article.

## Definitions and preliminaries

The speed prediction aims to obtain the future speed as accurately as possible over a specific time horizon. The essence of speed prediction is the identification of future traffic patterns, which describes the traffic operation mode and the relationship between the future speed and historical traffic states. Two important factors determine the prediction performance: the model and available information. The former takes charge of extracting patterns, which will be described detailed in Section 3-5. The latter, as the input of prediction model, is the foundation of prediction and can significantly affect the prediction accuracy. In addition, the features of information directly determine the information quality, such as data type, sources (i.e., fixed point data, vehicle trajectory data), format (i.e., scalar, vector, matrix), and processing (i.e., map matching, data cleaning). This article focuses on the type and amount of information utilized in speed prediction. This section begins with the definition of speed prediction in the transportation system, which explores the similarities and differences of speed prediction at different levels. Then, the influencing factors of speed are reviewed, and the main factors of the speed at different levels are analyzed.

### Definition of speed prediction at different levels

On the one hand, traffic flow exhibits periodicity and consistency. On the other hand, it is volatile and chaotic with the influence of contextual factors. Whether speed in the transportation system can be predicted and whether a pattern worth being captured exists are the basis of predictability. Predictability is defined as the possibility of achieving the desired prediction accuracy over a specific horizon (Yue et al., 2007). Overall, the speed at the proposed three levels is predictable, which is confirmed by a large amount of work on speed prediction (Wang and Shi, 2013; Lefevre et al., 2014; Gu et al., 2019b).

**Figure 1:**
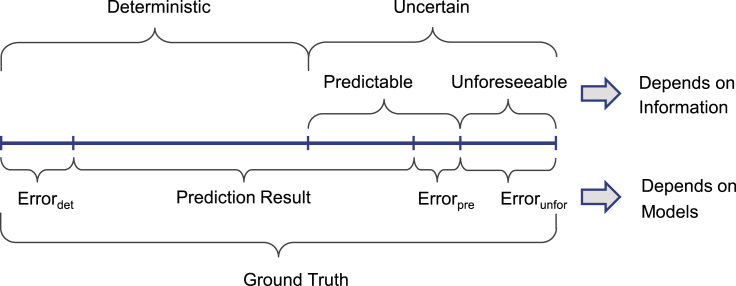
Components of a prediction variable

Given a prediction problem over a certain horizon, the ground truth can be divided into deterministic and uncertain part (Yue et al., 2007) as shown in Figure 1. In this article, the deterministic part refers to the trend and range of speed, which is determined by the available information. For example, the speed of an accelerating vehicle is likely to increase at the next moment and impossible to slow down instantly. Meanwhile, the uncertainty caused by contextual factors reflects the stochastic and chaotic characteristics of the traffic system. Parts of uncertainty are predictable, but the rest is unforeseeable depending on how well it is understood (Yue et al., 2007). Thus, the theoretically predictable value consists of the deterministic and the predictable portion in the uncertainty part (Nagy and Simon, 2018). The information basis determines the theoretically predictable value and determines the ceiling of prediction accuracy. In practice, the model used for prediction inevitably contains an error, as the three parts of error shows in Figure 1. Therefore, considering the various contextual factors it is essential to increase the theoretically predictable value, and the improvement of performance is the key to decreasing the error. In the context of predictability analysis, each speed prediction is defined as follows.

#### Traffic speed prediction

Traffic speed prediction is represented by the average speed of multiple vehicles passing through a specific road section over a period. The physical structure of road networks brings spatial and temporal dependency to traffic speed, which is key to improving prediction accuracy (Ma et al., 2017). However, how to model the dependency is still a challenge (Zheng et al., 2020).

##### Spatial dependency

**Figure 2:**
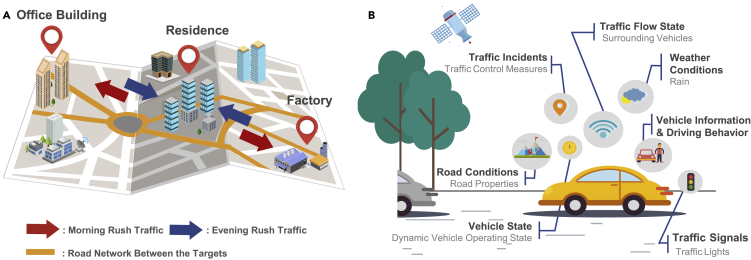
Traffic-speed prediction (A) and vehicle-speed prediction (B)

Traffic flows follow the road network, where the speeds of spatial points in the vicinity are correlated. Different spatial points have different effects on the prediction result and the spatial dependency at different time is different. The spatial dependency can be divided into local and global ones (Yin et al., 2021b). The former focuses on the local neighbor roads and the other concerns the connectivity of the entire network. In addition, a region in the road network is usually space-dependent with another through various non-Euclidean relation such as spatial adjacency, Point of Interest (POI) (Ge et al., 2019), and semantic information (Liao et al., 2018). As shown in Figure 2A, the three marked regions are connected and spatial adjacent to each other through the yellow road network. The POI attributes of the three marked regions also affect the corresponding traffic pattern.

##### Temporal dependency

The current speed at a specific point is correlated with the historical speed. Temporal dependency is complex non-linear and differs from one point to another. In addition, different time slices of speed bring different effects on the current speed (Wu et al., 2018). Moreover, traffic speeds are cyclical in the temporal dimension; for example, traffic speed is similar on weekdays and different from weekends. An important representation of this similarity on the weekday is the morning and evening rush, which is denoted by the red and blue arrows as shown in Figure 2A.

#### Vehicle speed prediction

Vehicle speed prediction focuses on a single vehicle as shown in Figure 2B. No direct spatial dependency is exhibited in vehicle speeds. Besides, the obvious uncertainty of vehicle behavior brings a more complex temporal dependency than traffic speed. Moreover, the prediction is also characterized by a short prediction horizon and strict real-time requirements, as vehicle speed prediction is mainly used for energy management (Zhang, 2019) and thermal management (Wang and Infield, 2018) of eco-driving.

#### Lane-level speed prediction

**Figure 3:**
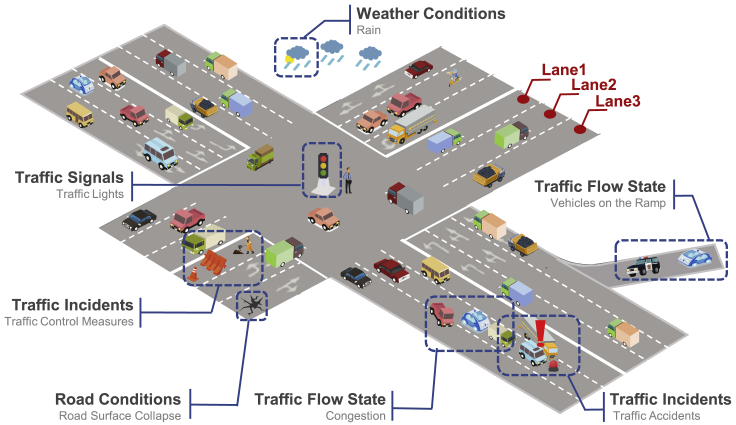
Lane-level speed prediction

Lane-level speed prediction aims to obtain the average speed of vehicles passing through a certain lane cross-section within a certain period. Figure 3 shows a brief description of the lane-level scenario. Unlike the macroscopic or microscopic speed prediction, the granularity of lane-level speed prediction is mesoscale (Ke et al., 2020a). The characteristics of this prediction lie between traffic and vehicle-speed prediction. Moreover, the modeling of interaction and spatio-temporal dependency at lane level are the challenges of this prediction.

### Influencing factors on speed prediction

In this article, factors influencing speed are summarized according to two principles: internal/external and static/dynamic. The former is classified by vehicle perspective, which represents the internal factors of the vehicle and the external factors of the environment, as shown in Figures 2B and 3. In the latter classification, static factors are constant and can be accessed online through offline storage. On the other hand, dynamic factors vary over time such that it is more difficult to model them than static ones. Thus, historical and current information of dynamic factors usually should be considered together in prediction. Finally, this section analyzes the main influencing factors of different predictions.

#### Internal factors

##### Driving behavior (dynamic)

The human driver directly operates the vehicle, and his/her driving behavior significantly affects vehicle speed. All the other factors affect the speed indirectly through driving behavior. However, the perception and decision of human driver are extremely complex (Lefevre et al., 2014; Lian et al., 2017). Drivers may be on the phone, talking, and becoming fatigued while driving. Furthermore, in the same scenario, the responses of different drivers are not similar, and even the same driver possibly makes different decisions (Yeon et al., 2019; Moser et al., 2015).

##### Vehicle information (static)

Vehicle information refers to the static factors about vehicle, including physical information (e.g., vehicle type, vehicle weight, passenger weight, and distribution), powertrain (e.g., power type, transmission type), power performance (e.g., maximum acceleration/deceleration, maximum speed, maximum climb), handling stability (e.g., minimum turning radius), etc. In addition, vehicle functions (e.g., ADAS) also belong to such information, which controls the vehicle speed according to specific rules.

##### Vehicle state(dynamic)

The above vehicle information constrains the change of vehicle speed indirectly, and the dynamic vehicle states directly affect speed. These factors describe the running state of vehicle, including vehicle speed, acceleration, available fuel or power (Sun et al., 2015a), battery temperature (Amini et al., 2020), transmission state (Li et al., 2018a), etc.

#### External factors

##### Traffic flow state(dynamic)

From a microscopic perspective, the traffic flow state refers to the moving state of surrounding vehicles, and the front vehicle states (i.e., relative speed and distance) heavily impact ego-vehicle speed (Yeon et al., 2019; Suh et al., 2020). From a macroscopic perspective, it refers to the traffic state, e.g., flow, speed, and occupancy (Liu et al., 2018a; Zhou et al., 2019a; Yan et al., 2018). For example, the traffic congestion can directly affect the speed at different levels, and the macroscopic shock waves are an influencing factor. In addition, the traffic flow state shows a strong spatio-temporal dependency, and the historical traffic flow state should be considered.

##### Weather conditions (dynamic)

The bad weather conditions (e.g., fog, rain, and snow) significantly affect the visibility and road friction, such that change the driving behaviors (Ahmed and Ghasemzadeh, 2018) and vehicle stability (Yang et al., 2020). For example, the low visibility caused by heavy fog or rain heavily impact the perception and control of vehicles (Ahmed and Ghasemzadeh, 2018; Fridman et al., 2019). Therefore, these factors may cause traffic congestion or accidents, which seriously affect the speed at different level.

##### Road and traffic rules (static)

Roads include urban roads, highways, rural roads and some functional roads such as ramps (Li et al., 2019b), intersections, transitional sections, sharp turns (Liu et al., 2018b; Qian et al., 2020; Yeon et al., 2019). The road properties (i.e., slope (Gu et al., 2019a), super elevation, curvature and roughness), environmental attributes (e.g., cluster, schools, hospitals) and traffic rules (e.g., speed hump, speed limits, lane properties) have an impact on speed (Shin and Sunwoo, 2019).

##### Traffic signals and events (dynamic)

Traffic events include traffic accidents (Xie et al., 2019a; Qian et al., 2020), traffic control measures, social events (e.g., sporting events, examinations, performances) (Polson and Sokolov, 2017), etc. The traffic accidents may lead to congestion, and the social events affect the speed by changing the traffic demand. In addition, traffic signals are the essential to keeping traffic in order on urban roads, and it severely constrains the speed in the transportation system (Zhou et al., 2019a). Furthermore, the lighting timing is dynamic thanks to real-time traffic management.

##### Factor analysis at different levels

All the above influencing factors impact the speed at different levels, but the main factors at each level are not the same. The micro speed refers to the vehicle speed such that the internal factors about the single-vehicle states are the main factors of vehicle speed in short-term prediction, especially the vehicle behavior. Besides, with the increase of prediction horizon, the impact of internal factors could decrease and the impact of external factors could increase thanks to their cumulative effect. With regard to the traffic speed, the effect of single-vehicle uncertainty on macro speed is attenuated by the statistical averaging over multiple vehicles. Therefore, compared to vehicle speed, the traffic speed varies steadily and the impact of internal factors on it is less. Meanwhile, the external factors become the main factors of traffic speed thanks to its longer prediction horizon than that of vehicle speed. As for lane-level speed, the impact of external and internal factors is between the macro and micro speed because of its mesoscopic perspective.

## Prediction methods of traffic speed

The spatio-temporal dependency is the main characteristic of traffic speed, such that it is the focus of prediction methods modeling. Meanwhile, integrating various influencing factors into model is the trend of prediction. Table 2 shows the summary and taxonomy of the method used for traffic speed prediction. We review the methods in terms of model-based, classical data-driven, and deep-learning methods. Meanwhile, the information utilized and how to model them is the concern of this section.

**Table 2:** Methods of traffic-speed prediction

Model type	Modeling traffic spatial dependency	Modeling traffic temporal dependency	Modeling external factors	Reference
Statistical Methods		ARIMA, SARIMA	–	Williams and Hoel, 2003; Lippi et al., 2013
	VARIMA, STARIMA		–	Chandra and Al-Deek, 2009; Min and Wynter, 2011
	–	Kalman Filters		Lippi et al., 2013; Tampere and Immers, 2007
Traditional Machine Learning	Probabilistic Graph	–	–	Furtlehner et al., 2021; Zhu et al., 2016 Rapant et al., 2016; Qi and Ishak, 2014
	Support Vector Machine		–	Yao et al., 2017; Asif et al., 2014; Wang and Shi, 2013
	–	Gaussian Process Method		Le et al., 2017; Chen et al., 2014; Lin et al., 2018
	–	ANN	–	Csikós et al., 2015; Tang et al., 2017
Deep Learning	–	GRU	–	Zhao et al., 2019b; Chen et al., 2021
	–	LSTM	–	Cui et al., 2018; Ma et al., 2015 Zhao et al., 2019a; Wang et al., 2019
	–	RNN	SAE (Weather Condition, Time Information & Traffic Accident)	Yu et al., 2017; Qu et al., 2021
	CNN	–	–	Ma et al., 2017
		Gated CNN	–	Liu et al., 2018a
		LSTM	–	Zang et al., 2019
		LSTM	Weather Condition & Time Information	Lv et al., 2018
		LSTM	MLP (Weather Condition & Road Feature)	Yang et al., 2020
		GRU + attention	–	Wu et al., 2018
	CapsNet	–	–	Kim et al., 2018
		LSTM	–	Ma et al., 2021
	SGCN	CNN	–	Diao et al., 2019
		LSTM	–	Shin and Yoon, 2020
		LSTM	Traffic Incidents & Time Information	Xie et al., 2019a
		TCN	Road Feature & POI	Ge et al., 2019
		GRU	–	Diao et al., 2019; Zhao et al., 2020b
		Seq2Seq (RNN)+Attention	–	Zhang et al., 2019
	DGCN	RNN	–	Chen et al., 2019
		Seq2Seq (GRU)	–	Li et al., 2018b
		CNN + Attention	The Other Traffic Properties	Feng et al., 2020
		TCN	–	Wu et al., 2019
	Attention	Attention	–	Park et al., 2020; Yin et al., 2021b; Zheng et al., 2020
		RNN	–	Zhang et al., 2018
	GCN + attention	RNN	–	Huang et al., 2020; Chen et al., 2020a
		CNN	–	Zhang et al., 2021a
	CNN + Attention	RNN + Attention	–	Wu et al., 2018
	RCNN + Errorfeedback		–	Wang et al., 2016
	RGCN		–	Wang et al., 2018; Guo et al., 2021
	GAT + TCN + Attention		–	Zhang et al., 2020a
	Road Network Topology + RNN		–	Wang et al., 2019; Kim et al., 2019; Lee et al., 2020

### Model-based methods

The model-based methods designed for traffic-speed prediction can be categorized according to the granularity as macroscopic, microscopic, and mesoscopic. The three levels are similar to the classification used to discuss the speed prediction problem in this article, but the specific definitions are different.

Macroscopic methods utilize macroscopic traffic properties (i.e. average speed, average traffic flow, and density) to describe traffic and analyze the traffic behavior through traffic flow theory. A classical macroscopic method is kinematic wave (Newell, 1993) which can reproduce the propagation of traffic waves. However, junctions are the bottlenecks of the kinematic wave method where multiple traffic waves meet. Reference (Jin, 2010) used the concepts of demand and supply to formulate the merging in kinematic wave and obtained analytical solutions. Meanwhile, the merging process was regarded as fair queuing based on their capacity in (Ni and Leonard, 2005). In addition, the choice of coordinate system in such methods directly affects prediction accuracy. Reference (Van Wageningen-Kessels et al., 2010) proposed a Lagrangian coordinate system that moves with the vehicle, resulting in a more accurate result than the traditional coordinate system.

Microscopic model-based methods aim to simulate traffic with the detailed responses of traffic participants via modeling their behavior. The intention of participant and interaction between each determine its behavior such that the Cellular Automata with the advantage of modeling the interaction is popular (Nagel and Schreckenberg, 1992; Maerivoet and DeMoor, 2005). Reference (Korček et al., 2011) eliminated the unwanted properties of Cellular Automata and applied the model in the speed prediction of large-scale networks. Considering the influence of special vehicles, reference (Zhao et al., 2020a) introduced some special rules for Cellular Automata(Zhao et al., 2020a), such as the low reaction time of connected-automated vehicle and regional avoidance of emergency vehicles.

The microscopic methods are computationally intensive due to detailed simulation, but they can describe the traffic in detail. On the contrary, macroscopic methods can effectively simulate road networks, but they are challenging to indicate participants' responses. However, the mesoscopic method combines different advantages of both macroscopic and microscopic methods (Chiu et al., 2010). A representative mesoscopic method is the gas-kinetic model (Helbing, 1996), which captures the dynamic vehicle behavior by probability distributions of traffic properties. Thanks to its mesoscopic level, such methods can achieve a better balance between efficiency and accuracy than the above methods.

In conclusion, model-based methods are difficult to guarantee the real-time requirement of traffic-speed prediction due to the complex traffic model, especially for the microscopic methods, even though they can provide interpretable results. Moreover, artificial assumptions and limited expertise can lead to inevitable errors in the face of dynamic traffic.

### Classicaldata-driven methods

Data-driven methods aim to inductively identify the traffic pattern behind the data and achieve prediction depending on the pattern. The statistical methods predict the future traffic speed primarily based on the temporal dependency of historical speed, of which Autoregressive Integrated Moving Average (ARIMA) is the most popular. ARIMA models the traffic speed as time series and combines autoregressive models, moving average models, and differentiation to capture the temporal dependency. Subsequently, more influencing factors are considered into ARIMA to improve accuracy. For example, SARIMA (Williams and Hoel, 2003) integrated seasonal feature into prediction, and VARIMA (Chandra and Al-Deek, 2009) considered spatial dependency features through upstream and downstream multi-points data. Further, STARIMA (Min and Wynter, 2011) took the limited spatio-temporal feature into account. However, ARIMA and its variants are computationally intensive as the number of relevant points increases. On the other hand, the Kalman filters (KF) are applied to traffic-speed prediction because they can estimate the dynamic system state from noisy data with good real-time performance (Lippi et al., 2013; Tampere and Immers, 2007). Moreover, other statistical methods, such as non-parametric regression (Clark, 2003) and partial least square (Li et al., 2020) are also employed in speed prediction. In addition, the different traffic properties are the contextual factors often considered by statistical methods. Reference (Yu et al., 2019b) used piece-wise function to capture the influence of traffic properties, such as traffic flow of adjacent roads. Meanwhile, the Jenks clustering with dynamic programming are adopted to determine the segment intervals. However, the statistical methods are too simple to extract the dynamic traffic patterns due to the inability to process various factors and stationary assumptions.

Therefore, traditional machine learning is employed in traffic-speed prediction, which can process high-dimensional information and extract complex traffic patterns, and it can be broadly classified into four categories.

#### Probabilistic Graph Method

*Probabilistic Graph Method* is the first category, which utilizes a graph to represent the joint probability distribution of the variables in the model. Markov Field is based on undirected acyclic graph (Furtlehner et al., 2021) and static Bayesian Networks (BN) is based on directed acyclic graph (Zhu et al., 2016). Besides, dynamic Bayesian networks are the basis of Hidden Markov Model (HMM) (Rapant et al., 2016). The probabilistic graph can effectively capture the traffic uncertainty, which is an obvious advantage of such methods. Reference (Zhu et al., 2016) integrated the speed categorical variable with spatio-temporal dependency modeling to improve the accuracy through BN because this method can consider continuous and discrete variables together. In (Rapant et al., 2016) and (Qi and Ishak, 2014), the HMM was applied to the speed prediction in freeways, where the dynamic changes of speed states are described by the transition probabilities, and its great robustness for noisy data was shown.

#### Support Vector Machine

*Support Vector Machine (SVM)* is the second category, which transforms the input space to a feature space by basis functions and applies a linear model in the feature space (Yao et al., 2017). SVM are binary linear classifiers by drawing the linear boundary in the feature space, and it aims to maximize the gap width. Therefore, SVM is essentially a problem of convex quadratic programming such that global optimization can be reached. Moreover, the nearest data point to the classification boundary is the support vector, which determines the computational complexity of SVM. In addition, the kernel function ensures the adaptability of SVM for non-linear problems, which transforms the inseparable data in the input space into separable data in the feature space, but the choice of kernel function is still a challenge.

SVM is widely used to extract spatio-temporal features, even the complex features of large-scale networks with different areas (e.g., urban and rural) (Asif et al., 2014). With regard to the kernel function, the common choice is standard functions, such as the Polynomial and Gauss radial basis kernel function, but it is necessary to redesign and adapt to the specific problem. For example, the wavelet function is employed to capture the non-stationary features of traffic speed in a novel kernel function (Wang and Shi, 2013).

#### Gaussian Process Method

*Gaussian Process Method* is the third category, which is a generalization of multivariate normal distributions in infinite-dimensional space. The relationship between *f*(*X*) and *X* can be expressed as a Gaussian process prior with the mean *m*(*X*) and the covariance function $k\left(x,x^{\text{′}}\right)$. Besides, the kernel function is the core of a Gaussian process which measures the distance between two sample points. The probability interpretation and CIs of output can be provided by non-parametric Bayesian formulation (Le et al., 2017) in Gaussian Process.

With regard to traffic-speed prediction, this method is mostly employed in the prediction with traffic information fusion, because it can take high-dimensional data, data heterogeneity, uncertainty, and ambiguity into account. For example, the weather information and traffic properties were considered in prediction through Gaussian process (Chen et al., 2014), and the social mediadata and car trajectory data were taken into account in the same way in (Lin et al., 2018). However, the cubic learning computation and quadratic space requirement are the major limitations of the Gaussian process (Le et al., 2017).

#### Artificial neural network

*Artificial neural network (ANN)* is a weighted computational network consisting of several layers of neurons (computational cells), which is inspired by the neural in the biological brain (Csikós et al., 2015) and has been a popular method in traffic-speed prediction thanks to the great robustness. Research (Csikós et al., 2015) used ANN to predict the traffic speed of urban networks. Moreover, a fuzzy ANN was proposed in (Tang et al., 2017), which combined the advantages of fuzzy inference and neural network: knowledge expression and learning ability. Research (Huang and Ran, 2003) inputted the adverse weather information into ANN to consider the impact of weather factor.

### Deep-learning methods

Compared to traditional machine learning with shallow structures, deep learning methods can not only handle large-scale data but can also extract the complex pattern between multiple factors and traffic speed. This section discusses the deep learning methods in terms of spatial dependency, temporal dependency, spatio-temporal dependency, and external factors modeling. Based on the generational classification of deep-learning methods for traffic prediction in (Lee et al., 2021), this article does not focus on the first generation methods (i.e., Deep Belief Networks and Stacked AutoEncoder (SAE)) but pays attention to the latest deep learning methods.

#### Spatial dependency modeling of traffic-speed

##### CNN

The Convolutional Neural Networks (CNN) are a series of models to process images inspired by the human visual nervous. The convolutional layer applies several convolutional kernels to extract different local features of the image. The pooling layer further reduces the data dimensionality and captures the spatial dependency of different local features. Finally, the fully connected layers output the results based on the features. Moreover, parallel computation is a crucial advantage of CNN thanks to the independence of convolution operations, which can facilitate the training significantly with the weight-sharing mechanism. The traffic speed data in road networks can be modeled as an image and learned by CNN (Tedjopurnomo et al., 2020), and the spatio-temporal matrix was usually used to convert traffic to image (Ma et al., 2017; Wang et al., 2016), where the matrix elements represent speed values in the corresponding position and time.

Thus, it is suitable for spatial dependency modeling and outperforms traditional machine learning even Recurrent Neural Networks (RNN) (Ma et al., 2017). Moreover, researchers have proposed some variants to consider more influencing factors than CNN to improve prediction performance. Reference (Wang et al., 2016) introduced the individual neurons with error feedback mechanisms into CNN to cope with the challenges arising from traffic emergencies, such as traffic peaks and accidents. Concerning long-term prediction, research (Zang et al., 2019) fed the traffic data with different time scales to prediction model and used four CNN modules to extract multiscale spatial features.

##### CapsNet

Despite these considerable advantages of CNN, the pooling operation in CNN results in some important information (i.e., feature locations and their relative spatial relationships) loss and ridiculous results. For example, CNN usually recognizes a human faces with misplaced face features as a normal face. Therefore, the Capsule Network (CapsNet) has recently received a large amount of attention, which replaces the pooling operation with dynamic routing and extracts more accurate spatial relationships among the road segments (Kim et al., 2018). Considering the interrelationship of topology, CapsNet is suitable for large-scale prediction. Reference (Kim et al., 2018) adopted CapsNet to capture the interrelationship of traffic networks in different time steps, which showed a better result than CNN. Reference (Ma et al., 2021) integrated various spatial features (e.g., position, direction, length) of traffic networks into the capsule vectors and extracted comprehensive spatial dependency. However, the training of CapsNet consumes more time than CNN due to its more complex construct.

##### GCN

The traffic speed is described as an image structure to extract spatial dependency in CNNs, but the spatial structure of the traffic network is non-Euclidean in essence, where the relations between road nodes are different. For example, the number of nodes connected to each junction node is not the same, and the road properties of each node are various. Due to such characteristics, it is not feasible to utilize the same size local convolution kernel for all nodes directly such that the convolution kernel of CNN is no longer suitable for non-Euclidean data. Therefore, Graph Convolutional Network (GCN) designed for non-Euclidean data have attracted extensive interest, which can be roughly classified into two categories.

Spectral GCN (SGCN), as the first category, defines the convolution in the spectral domain and transfer traffic data to this domain by graph Fourier transforms (Bruna et al., 2014). Because SGCN can simplify the computation and transform the convolution in spatial domain to a product in frequency domain. From the perspective of signal processing, the graph convolution is regarded as a noise filter (Yin et al., 2021a). Research (Zhao et al., 2020b) used the filter to capture spatial features between the nodes by its first-order neighborhood, then built GCN by stacking multiple convolutional layers. SGCN investigated the traffic graphs through the eigenvalues and eigenvectors of the Laplacian matrix, which is the difference between the diagonal matrix and the adjacency matrix (Diao et al., 2019). The adjacency matrix of graph structure determines the performance of GCN such that it is one of the research focus. Most existing studies use a single measure to construct the adjacency matrix, leading to an inadequate description of spatial dependency. Therefore, the Multi-Weight GCN considered specific structural features (i.e., speed limit, distance, and angle) in the adjacency matrix and weighted them to describe dynamic spatial dependency. In addition, research (Diao et al., 2019) designed a deep-learning Laplacian matrix estimator to update the real-time matrix and capture the change of traffic pattern.

The other one defines graph convolution based on the spatial structure between graph nodes (Atwood and Towsley, 2016). The diffusion GCN (DGCN) is the most popular that regards graph convolution operation as a diffusion process. Such methods assume the information is transmitted from one node to adjacent nodes with a transition probability such that it can reach a dynamical equilibrium after a few steps. Based on the assumption of state transition, DGCN models the spatial dependency as a stochastic dynamic process, unlike the fixed structure of SGCN. In addition, it generally adopts the bidirectional diffusion mechanism to capture the complex spatial dependency. Research (Li et al., 2018b) integrated DGCN into the Sequence to Sequence (Seq2Seq) to extract the spatial dependency in multi-step speed prediction. To improve the model adaptability for different scenario, research (Wu et al., 2019) proposed a self-adaptive adjacency matrix that can learn unknown graph structures automatically, and the multiple spatial layers were stacked to capture the spatial dependency at different temporal levels.

##### Attention

The attention mechanism is inspired by the attention of human vision, and its core is a set of attention allocation coefficients. In respect of spatial dependency, the attention mechanism can explicitly highlight the roads with a high impact on speed by learning different road unit weights. The most popular work is graph attention networks (GAT) (Pan et al., 2019; Chen et al., 2020a; Huang et al., 2020), which aggregates features from neighboring nodes to the central node by the attention coefficients. Study (Zheng et al., 2020) adopted an attention mechanism to extract temporal and spatial dependency, and proposed a gated method to fuse the features from the attention model (Zheng et al., 2020). Moreover, multi-head attention can capture the features in different subspaces and improve the prediction accuracy, which has lately received great attention. However, traditional multi-head attention treats each head equally. Research (Zhang et al., 2018) further designed a convolutional sub-network to learn the weights and improve the effect of attention. Considering the dynamical change of spatial dependency, the spatial attention module with sentinel vectors are introduced in (Park et al., 2020), which can dynamically adjust the spatial features based on road states. Research (Yin et al., 2021b) proposed an attention mechanism to model the coupling correlations among heterogeneous data. Meanwhile, it divided traffic nodes into different sets based on their adjacency relations and capture the dynamics of different nodes, even global spatial dependency. In respect of data security, research (Zhang et al., 2021a) proposed a differential privacy-based approach and its aggregation mechanism for adjacency matrix, which achieves the trade-off between privacy and performance.

#### Temporal dependency modeling of traffic-speed

##### RNN

RNNs are a class of learning networks designed for sequential data. Different from ANN, each calculation step of RNNs will return the result as an input, and the result of the next step is determined by the new input and previous result. Therefore, RNNs are able to consider the influence of the previous input on the following output with the help of this circular structure. The common format of traffic data is sequential time series, thus RNNs are the ideal candidate for extracting temporal dependency. However, the classical RNN suffers from the attenuation or explosion of network gradients caused by the gradual decrease or increase of parameters in the cyclic computation, leading to the inability for long-term memory in sequences. Moreover, the optimal time lag of RNN needs to be predetermined and mainly is based on a trial-and-error approach.

In an effort to overcome this challenge, researchers proposed Long Short Term Memory Networks (LSTM) by adding a gate control unit to RNN hidden layer. The gates of LSTM are forget, input, and output gates, which selects and store the important information in each step to consider long-term information. As a result, LSTM has been applied more often in speed prediction compared to RNN (Shin and Yoon, 2020; Ma et al., 2015; Cui et al., 2020; Xie et al., 2019a; Niu et al., 2019; Bogaerts et al., 2020). Research (Zang et al., 2019) focused on the long-term prediction at least 24 h and stacked three ConvLSTMs to extract the multiscale temporal features. In addition, the bidirectional mechanism is a significant improvement of classical LSTM (Cui et al., 2018; Wang et al., 2019), which considers the temporal features in both forward and backward directions. Moreover, data quality directly impacts prediction accuracy. A data cleaning rule (Zhao et al., 2019a) and a filling method of missing data based on trend-historical data was adopted before the prediction by LSTM. Research (Tang et al., 2020) constructed estimations for missing values before the predicting by LSTM, which capture global temporal dynamics for missing data and adversarial training to enhance the modeling of global temporal distribution.

Although LSTM can overcome the problem of classical RNN, its three gates structure leads to high computational cost. Therefore, a simple RNN variant, Gate Recurrent Unit (GRU), receives great attention, which simplifies the gate control units of LSTM to two gates (reset gate, update gate) and can achieve similar performance to LSTM in practice (Zhao et al., 2020b; Li et al., 2018b). The bidirectional mechanism can be also integrated with GRU, and research (Chen et al., 2021) adopted the bidirectional GRU to extract the multiscale temporal dependency based on the multiscale-grid model. Moreover, parameter optimization can directly affect prediction performance. Reference (Zhao et al., 2019b) proposed a GRU with weight optimization for urban express, and showed the great performance of Rmsprop algorithm. Meanwhile, Bayesian optimization is regarded as an effective approach for parameter optimization in (Zhang et al., 2020b). In addition, the Encoder-Decoder structure, usually consisting of the RNNs, is employed to handle the issue of different lengths of input and output sequences (Li et al., 2018b; Zhang et al., 2019, 2020d). However, the limitation of Encoder-Decoder is obvious. No matter what the length of the input and output, the intermediate vector length is fixed. The issue usually results in a loss of information, and it could become worse as the length of the sequence increases.

##### Attention

The attention mechanism can extract the key parts of inputs rather than encode all the information into vector input. In order to capture dynamic temporal dependency, the attention mechanism has attracted extensive interest (Park et al., 2020). For example, reference (Wu et al., 2018) used the attention model to extract the salient part of traffic data and achieve multi-step prediction. To model the multiscale temporal dependency, research (Feng et al., 2020) designed an attention model to extract whole-range global temporal features and used stacked dilated convolution to capture local temporal features.

##### CNN

Despite RNN is popular in temporal dependency modeling, but it suffers from a high computational cost. On the contrary, CNN can facilitate the training by parallel computation. Thus, research (Diao et al., 2019) captured the short-term temporal features by a 2D temporal convolutional layer. Moreover, the gating mechanism is a common improvement for CNN. The gated linear units' convolution were employed in both short and long-term prediction (Huang et al., 2020), which simplified the network layers to reduce the accumulated errors in long-term prediction. Research (Liu et al., 2018a) adopted an attention model to weight the feature maps and channels, then extracted temporal features by Gated CNN.

##### TCN

The classical CNN is unable to capture long-term temporal dependency well due to the convolutional kernel size. Therefore, it is generally regarded as an unsuitable method for sequential data compared to RNN. However, CNN has the advantage of computation such that a variant of CNN, Temporal Convolutional Network (TCN), is proposed for modeling sequential data. The size of local input space could affect the convolution result, and is referred to to the receptive field. To expanding the size of receptive field, dilated causal convolution, as the core of TCN, uses the strategy of interval sampling for input, where the receptive field size could grow exponentially with the increase of layer number. Therefore, TCN can obtain a long receptive field with fewer layers, and becomes a candidate for temporal dependency modeling. Besides, TCN is developed from CNN and overcomes the major issue of gradient attenuation or explosion in RNN. The experiment results in (Zhang et al., 2020a) have demonstrated that TCN outperforms RNNs in both accuracy and computation time. Research (Wu et al., 2019) not only integrated gating mechanisms into TCN but also stacked multiple learning layers with TCN to capture the temporal dependency at different temporal levels. Furthermore, the residual connection in TCN allows the model to pass information across layers and reduce the training complexity. Several residual blocks were stacked in TCN to extract the feature of temporal dependency in (Ge et al., 2019) and a suitable number of residual blocks can improve the prediction accuracy.

#### Spatio-temporal dependency modeling of traffic-speed

Most of the existing research extracted the temporal and spatial features separately, and then fuse them by specific methods, such as concatenation, linear transformation, attention mechanism (Xie et al., 2020). The mainstream of spatial dependency modeling is convolutional methods and the popularity of temporal dependency popularity is sequential models. The simple method for capturing spatio-temporal dependency is to concatenate the methods used to extract different features, of which the group of RNN and GCN is the most popular (Wang et al., 2018; Guo et al., 2021). Besides, some researchers proposed an additional module for this problem. Research (Zhang et al., 2020a) used GAT and TCN to extract the two features separately, and further adopted multi-head self-attention to capture the spatio-temporal coupling effects. However, the temporal and spatial features are not independent in essence. The spatial dependency is different at different time steps, and the spatial structure is also an essential factor of temporal dependency. The neglect of interrelationship between the spatial and temporal features is an obvious limitation of the above method, and the deep-learning methods become the promising direction for this issue thanks to its excellent performance in feature extraction. Research (Xie et al., 2020) improved the DGCN to capture the spatio-temporal dependency, where the information was diffused simultaneously to the neighborhood and the next temporal state. In this way, heterogeneous spatial-temporal structures can be modeled as a homogeneous process of diffusion. Another research integrated the topology information of traffic networks into the deep-learning network (Wang et al., 2019) to capture spatio-temporal dependency. In (Kim et al., 2019), the topology of the traffic network was modeled as a spatio-temporal graph and integrated into RNN to model the complex dependency. Research (Lee et al., 2020) also projected the topology of traffic networks to construct the learning network, and enhanced the model interpretability.

#### External factors modeling of traffic speed

The spatio-temporal dependency modeling is the focus of the existing works, but it is necessary to consider the external influence factors causing random traffic fluctuations. These factors can be combined with the spatio-temporal learning network or be captured by an additional module. The weather condition and time information are usually considered together. Research (Lv et al., 2018) adopted two fully connected layers to capture the weather and time features. The first layer aimed to extract the holiday and weather features, and the other was used to map features to high dimensions. A Multilayer Perceptron (MLP) was used to capture the features of road properties, weather, and air quality in (Yang et al., 2020), and the traditional traffic theory was applied in the feature merge layer. Reference (Qu et al., 2021) fed the weather and time information as a feature matrix, and an Autoencoder is employed to extract the features in parallel.

The road feature and POI are important factors of spatial dependency. Research (Ge et al., 2019) used POI and the pairwise similarities of graph node to construct the adjacency matrix input. A traffic dataset with various information was released in (Liao et al., 2018), and Seq2Seq was adopted to extract the feature of offline geographical properties, social attributes, and road intersection information.

Urban traffic incidents and social events can reflect the dynamics of the traffic system (Xie et al., 2019a), and such information can be adopted to improve the robustness and adaptability of models. Research (Yu et al., 2017) proposed a DeepLSTM with stacked autoencoder to extract the accident features and jointly model the peak-hour and post-accident conditions traffic. Besides, the impacts of various incidents are different in essence such that research (Xie et al., 2019a) designed a method to discover the traffic incidents with high impact on speed to improve prediction accuracy. Then, a learning network with two fully connected layers was adopted to extract the incident features. In addition, some works integrated traffic properties influence to improve prediction accuracy. Reference (Liu et al., 2018a) constructed the input as 3D matrices by traffic flow, speed, and occupancy to capture the features of traffic properties and improve the robustness for missing data.

#### Limitation

The deep-learning methods are able to extract diverse traffic features with its superior performance, and have led the research of speed prediction. In the face of the research wave, it is important to make sober and fair assessments of its benefits and drawbacks, and rationally explore the value of such methods.

First, deep-learning methods need to feed large amounts of data, and the data of some important scenarios are difficult to collect, such as accident scenario. Meanwhile, the training cost becomes high due to the heavy data demand and complex model structure. Meanwhile, the computational complexity could increase rapidly as more factors are considered. In addition, due to the black box characteristic, the interpretability of such methods remains a huge issue compared to classical methods. In conclusion, no one method is the panacea, and the deep-learning methods are the right choice only if their unique capabilities are worth its cost (López Manibardo et al., 2021).

## Prediction methods of vehicle speed

**Figure 4:**
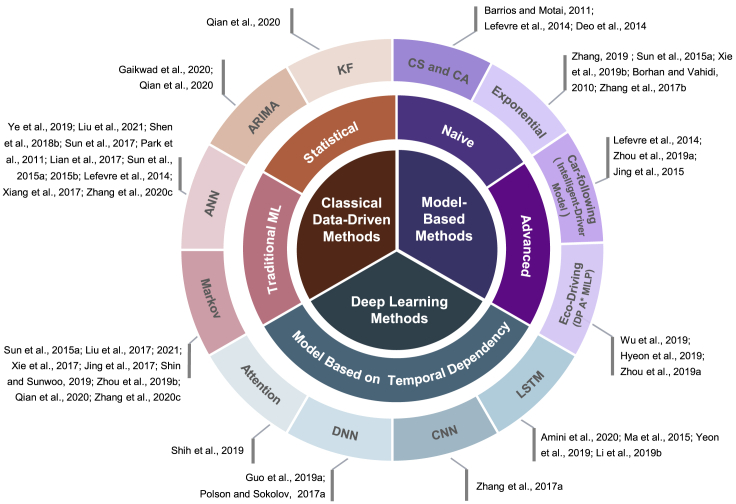
Vehicle-speed prediction methods ANN refers artificial neural network, KF refers Kalman filter, CS refers constant speed model, CA refers constant acceleration model, DP refers dynamic programming, MILP refers mixed integer linear programming, A∗ refers A-star algorithm, LSTM refers long short-term memory network, CNN refers convolutional neural network, DNN refers deep neural network, Attention refers attention mechanism

Prediction methods of vehicle speed can be also divided into model-based, classical data-driven, and deep-learning methods as illustrated in Figure 4. It has to be noticed that vehicle speed is the most uncertain variable at different levels. On the one hand, the vehicle speed is directly affected by its internal factors, and it is difficult to model the complex driving behavior. On the other hand, external factors also impact vehicle speed indirectly. In addition, the spatial dependency of vehicle speed is not very obvious without the networks as a reference. Because of its shortest prediction horizon and real-time requirement among different levels, it is crucial to adopt a less complex model in vehicle-speed prediction. Meanwhile, the adaptability of those prediction methods is more essential than the speed prediction at other levels, because of the various vehicle condition and uncertainty of driving behavior.

### Model-based methods

The model-based method is interpretable and simple to realize such that it is suitable for vehicle-speed prediction. According to the proposed assumption, model-based methods can be divided into naive and advanced models (Yeon et al., 2019).

#### Naive model-based methods

The naive model usually used simple rules of vehicle speed or acceleration to describe the vehicle’s motion. Such models are only based on real-time vehicle information and can be divided into three classifications: constant speed model, constant acceleration model, and exponential model.

##### Constant speed (CS) and constant acceleration (CA) models

CS model assumes that the vehicle maintains the same speed while driving,

(Equation 1) a(t+Δt)=0

The CA assumes that the vehicle will maintain the same acceleration,

(Equation 2) a(t+Δt)=a(t)

The CS model is very popular in collision prediction (Polychronopoulos et al., 2007). It is the basis for calculating the time to collision for risk assessment (Vogel, 2003). CA model, like the CS, is also used in short-term predictions such as collision prediction (Barrios and Motai, 2011). It is also suitable for accurately predicting the motion state of smart devices to achieve positioning functions. Meanwhile, CA and CS models can reliably predict the movement of surrounding vehicles to plan the trajectory of autonomous vehicles (Deo et al., 2018; Wang et al., 2003).

##### Exponential models

Unlike the CA and CS, the exponential model assumes the future speed varies according to a more complex law (i.e. exponential) than CA and CS. Such methods are also based on the current speed information only. Set $t_{p}$ as the prediction time horizon and $t_{k}$ as the time step increasing from oneto $t_{p}$. The relationship of the speed of the transition from time *t* to time $t+t_{k}$ can be expressed by an exponential function, that is,

(Equation 3) $$v_{t+t_{k}}=v_{t}{\left(1+\varepsilon \right)}^{n},t_{k}\in 1,2,\cdots ,t_{p},$$

where*v*_*t*_ is the speed at time *t*, $v_{t+t_{k}}$ is the speed obtained by predicting the *t*_*k*_ second backward at time *t*, and ε is the exponential coefficient.

Furthermore, some researchers assume the torque demand varies according to an exponential law, and the change in torque over time results in fluctuations of speed. Research (Borhan and Vahidi, 2010; Xie et al., 2019b; Zhang et al., 2017b) used the relationship between torque and speed to achieve prediction.

#### Advanced model-based methods

The key of model-based methods is to model the driving behavior, which reflects how the driver response to the environment. Naive models usually only take less internal factors of the vehicle into account and oversimplify the driving behavior. With the development of autonomous vehicles and V2X, advanced models (i.e., car-following model and eco-driving model) have received great attention, which can model a variety of external factors, such as traffic light information, surrounding vehicle information. The car-following model focuses on the interaction with the front vehicle. Based on the speed and position information of the leading vehicle, the intelligent driver model was used in (Jing et al., 2015) to describe the vehicle behavior and estimated the time headway parameter for speed prediction. However, this model only considers the behavior of car-following, which limits the result accuracy, especially in long-term prediction. Therefore, to save vehicle energy in long term, research (Zhou et al., 2019a) planned the vehicles' trajectories according to the eco-driving rules with the information of traffic lights and surrounding vehicles, and obtained the future speed of ego-vehicle from its trajectory directly. Furthermore, the improvement of accuracy means the increase of computation cost, and the V2X information requires the corresponding devices. Therefore, real-time and information availability should be considered in the advanced model.

### Classical data-driven methods

The data-driven methods do not require any calibration of the theoretical model and show better accuracy than model-based methods. Classical data-driven methods also can be classified into statistical methods and traditional machine learning methods.

#### Classicalstatistical methods

Classical statistical methods often are applied to a short-term prediction, mainly including ARIMA and KF. In (Guo et al., 2019b), ARIMA is used to predict vehicle speed for energy management, which is more accurate than many other linear methods. However, the temporal dependency of vehicle-speed prediction is too complex to be effectively captured by the ARIMA model (Liu et al., 2019). Meanwhile, these methods show poor adaptability for a dynamic driving environment. Therefore, hybrid statistical methods receive attention, which uses the complementary of different methods. Research (Qian et al., 2020) used the ARIMA model to predict the vehicle acceleration and input the time-varying result to KF to reach more accurate result. Meanwhile, it proposed an adaptive forgetting factor in KF to improve the filtering accuracy and real-time performance.

#### Traditional machine learning

##### Markov

Such a method can predict the state changes at future moments according to the current state based on Markov theory (Logofet and Lesnaya, 2000). A Markov process is a random process that undergoes a transition from one state to another, and the probability distribution of the next state only depends on the current state instead of the previous sequence (Wang and Infield, 2018). The dynamic driving process can easily be modeled as a Markov process based on the acceleration and speed state transition, and the driving uncertainty can be considered by its simple model structure. Research (Chib, 2001; Sun et al., 2015a) described the driving process by the random Markov model, and uses various cycles to train the state transition matrix. However, the difference between cycles is usually ignored, which could lead to inaccurate state transition and the deviations of prediction result. Therefore, research (Liu et al., 2017) used the neural network to choose the state transition matrix in different driving cycles before the random Markov method. Fuzzy encoding was also used to divide the acceleration events into multiple states (Jing et al., 2017), and the acceleration states are predicted by Markov transition. Besides, the increase of available states in Markov method brings the curse of dimensionality such that some research (Xie et al., 2017) used Monte Carlo approach to obtain the possible future state of Markov chain by sampling in the stationary distribution. Furthermore, it is difficult for a single Markov chain model to use multiple inputs, which limits its accuracy, such that multiple and self-learning Markov chain models receive the attention. Research (Liu et al., 2021) used historical traffic data to predict a random Markov transition probability, and adopted a neural network to learn the current speed information for prediction. Compared to the traditional Markov model, the self-learning Markov model does not rely on the offline training database to estimate the transition probability matrix, which improves its adaptability under different driving scenarios (Zhou et al., 2019b).

##### ANN

ANN is a popular method for vehicle-speed prediction, especially for long-term prediction (Lefevre et al., 2014), which usually outperforms the Markov and model-based method (Sun et al., 2015a). Meanwhile, the shallow structures of ANN become its advantage compared to the deep-learning methods in vehicle-speed prediction such that a trade-off between accuracy and computation cost can be reached. The first type of ANN is Radial Basis Function neural network (RBF-NN) (Sun et al., 2015b), which can approximate optimal functions with a faster convergence rate than the classical Back Propagation neural network (BP-NN). Research (Sun et al., 2017) inputted the information of driving conditions into RBF-NN and improves the model adaptability under different scenarios. Besides, to capture the speed dynamics, the non-linear autoregressive (NAR) neural network also is employed in vehicle-speed thanks to its dynamic characteristic, which can automatically integrate the output into the input to calculate the next output. Considering the impact of driving behavior on vehicle speed, research (Xiang et al., 2017) used the stroke of accelerator and brake pedal to identify current driving behavior first, and research (Lian et al., 2017) adopted fuzzy inference to identify driving behavior. Then they employed NAR-NN to predict speed under different driving behavior. Furthermore, other methods are usually integrated with ANN to reach an accurate result. Research (Zhang et al., 2020c) proposed a hybrid method consisting of the Markov method and BP-NN, which adopted the Markov method to grasp speed trend and helped BP-NN overcome the local optimal solution.

##### Integration with traffic-speed prediction

Although the definition of speed at different levels is not the same, all of them aim to describe the traffic, just with different perspectives. Speed prediction at different levels present close connections such that it is meaningful to combine them to improve prediction accuracy. The prediction results of traffic-speed are usually regarded as an important reference for vehicle-speed prediction, which reflect the dynamic traffic at micro level (Park et al., 2011). Research (Suh et al., 2020) first employed traffic flow model to predict the traffic state, and the results were used to extract the future position and speed of target vehicle. Analogously, research (Jiang and Fei, 2017) first utilized ANN to predict the traffic speed of road segments, and then adopted HMM to capture the statistical relationship between speed at macro and micro level.

### Deep-learning methods

Deep-learning methods can capture the complex patterns behind big data and usually obtain more accurate results than the other methods. Meanwhile, the disadvantages, such as high computing and storage cost, are prominent under the real-time requirement of vehicle-speed prediction. However, deep-learning methods are still the trend of vehicle-speed prediction, and it is necessary to continue further research. Unlike the spatio-temporal dependency of traffic speed, temporal dependency is the main characteristic of vehicle speed. Therefore, LSTM, as the mainstream of temporal dependency modeling, is suitable for vehicle-speed prediction. Reference (Liu et al., 2019; Gaikwad et al., 2020) has shown that LSTM can increase the result accuracy and decrease the transient time lag of this prediction compared to the above methods. Research (Yeon et al., 2019) used LSTM to capture the impact of ahead vehicle information, ego-vehicle states and location. This research also showed that the internal vehicle states, radar sensor information, and ego-vehicle location are the essential factors for vehicle speed prediction. Research (Li et al., 2019b) used the Pearson correlation coefficient method to discover the factors with high impact, and then the LSTM was combined with BP to model the temporal dependency. Besides, to especially capture the impact of driving behavior, research (Shih et al., 2019) proposed a hybrid model consisting of coders, LSTM, and attention model. Among them, the attention model is used to identify the start of the driving behavior. Then the especial seq2seq prediction model for different driving behaviors is selected to obtain the future vehicle speed. In addition, CNN is also employed in vehicle-speed prediction, and research (Zhang et al., 2017a) adopted CNN to predict the vehicle speed in different time horizons with the information of the leading vehicle (i.e., its speed and distance to the next traffic light).

## Lane-level speed prediction

Every vehicle running on the road is constricted by lane boundaries. The interaction of traffic flow between lanes significantly affects the speed of each vehicle. It is necessary to provide the lane-level speed information for each lane under the perspective of meso. The research on lane-level speed prediction has received attention only in recent years. Therefore, this section primarily elaborates the description and meaning of lane-level speed prediction and reviews the prediction methods at the meso level.

### Description of lane-level speed prediction

**Figure 5:**
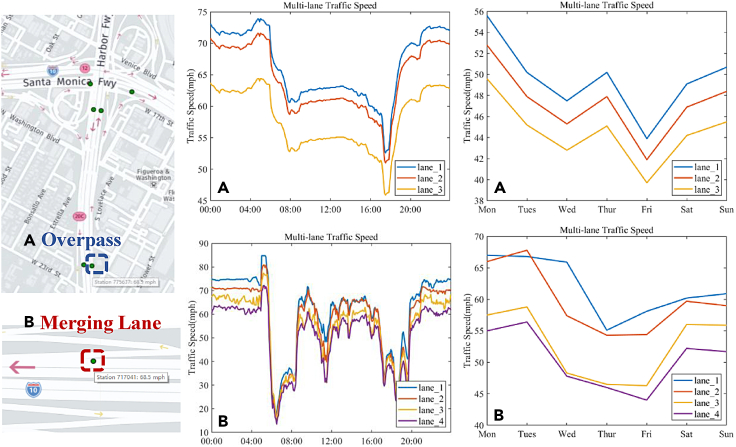
Lane-level average speed during a day and a week before the overpass and merging lane

Compared to the traffic-speed prediction, the lane-level speed prediction releases the assumption that the prediction methods of traffic-speed take the same traffic pattern for multiple lanes on the road. On the one hand, different lanes of the same road have different functions and restrictions thanks to their different locations. For example, the lanes on the China highway are called the overtaking lanes, carriage lanes, and emergency lanes from inside to outside. The speed limit of overtaking lanes is the highest, the occupancy of emergency lanes usually is prohibited. Those characteristics finally result in different lane traffic patterns. On the other hand, because of their interaction, the lane traffic patterns are correlative with the patterns of the other lane on the same road. The real traffic data can demonstrate the difference in lane-level speed. As shown in Figure 5, this article represents the lane-level speed during a day and a week before the overpass and merging lane. The data source is the dataset PeMS (Chen et al., 2001), and the sampling time of the week-speed data is 12:00 of each day. The observation points of the two scenes, which were marked by the dotted frame in the figure, detected the average speed of different lanes simultaneously. Taking scenario A as an example, there were three lanes at the observation point, i.e., lane 1, 2, and 3 as shown in Figure 3. The difference in lane-level speed between them can be up to 15.6% for a day before the overpass and 28.9% for a week before the merging lane. Meanwhile, the trend of different lane-level speed is similar. Therefore, the traffic patterns of different lanes on a specific road are different but correlative with the neighbor.

Lane-level speed prediction can capture different traffic patterns between different lanes and describe vehicle behaviors at meso level (Ke et al., 2020a). Therefore, the spatio-temporal features of lane-level traffic and interaction between lanes bring challenges and opportunities to this prediction. With regard to the application of lane-level speed prediction, it not only provides fine granular future traffic states for traffic management, but also helps the vehicle select a suitable lane and plan the optimal driving path.

### Prediction methods of lane-level speed

Lane-level speed prediction is a new research field as compared to traffic and vehicle-speed prediction. Nowadays, the deep-learning method has become the mainstream of speed prediction. Therefore, the existing research on lane-level speed prediction focuses on this method. The lane-level traffic speed is more difficult to process than the speed at other levels due to the dimension of lane. To simplify the problem, a popular method is to divide the data according to the time or lane. Research (Raza and Zhong, 2017) considered the peak or off-peak conditions and utilized the genetic algorithm to optimize the prediction parameters. Then, the ANN and local weighted regression model are adopted to predict the lane-level speed in different conditions. Research (Tao et al., 2020) used an attention model to extract the important feature and assigned the weight according to different lanes and times. In addition, the multi-channel spatio-temporal image are usually employed for lane-level speed, which can represent the information of temporal and spatial dimension along the corridor as well as the space information across lanes. Processing image data is the strength of CNN such that CNN (Ke et al., 2018) is the preference for this prediction with multi-channel image input. Besides, the characteristic of traffic properties are similar such that the multi-channel image are able to consider the impact of traffic properties. The model construction (Ke et al., 2020b) is similar to that in (Ke et al., 2018), and it converted the lane-level speed and flow data into a spatial-temporal matrix with multi-channel to input CNN.

With regard to modeling the spatial-temporal dependency at lane-level, the common approach is to extract the lane-level temporal and spatial features separately (Ke et al., 2018,2020b; Raza and Zhong, 2017; Tao et al., 2020), but it ignored the inherent relationship between temporal and spatial features. Research (Lu et al., 2020a) introduced the Conv-LSTM structure to extract spatio-temporal dependency efficiently and simultaneously, and the multi-channel image is suitable to describe the spatio-temporal dependency. Moreover, it is crucial to consider the trade-off between efficiency and accuracy of prediction. Therefore, some research designs a module to discover the feature with high impact before prediction. Research (Lu et al., 2020c) adopted random forest method to extract the importance of the temporal dependency and simplify the input, and then used clockwork RNN to capture the temporal features. Besides, study (Gu et al., 2019b) extracted the spatial features by entropy-based gray relational analysis and selected the lane section with the greatest impact by the correlation analysis methods. The training process in (Gu et al., 2019b; Lu et al., 2020c) was accelerated in the time dimension and space dimension, respectively.

## Evaluation

This section begins with the evaluation metrics of speed prediction and presents the public datasets and open-source codes. Finally, the challenges and future directions of speed prediction are discussed in detail.

### Evaluation metrics

It is necessary to evaluate the performance of the speed prediction model for comparing and developing them. The available metrics can be summarized as four categories.

#### Prediction horizon

The time horizon of prediction, as an important prerequisite, directly determines the characteristics of prediction problem. Meanwhile, the predictability and accuracy generally decrease with the increasing of prediction horizon (Yue et al., 2007).

#### Absolute error metrics

To evaluate the prediction accuracy, researchers have proposed a series of metrics based on the absolute error between the prediction result and ground truth. Mean absolute error (MAE) and Mean absolute percentage error (MAPE) aim to evaluate the relative bias, and Variance APE (VAPE) denotes the dispersion of absolute error (Yang et al., 2017).

(Equation 4) $$MAPE=\frac{100\text{\%}}{MN}\sum_{j=1}^{M}\sum_{i=1}^{N}\frac{\omega_{Ai}^{j}|\hat{y_{i}^{j}}-y_{i}^{j}|}{y_{i}^{j}},$$

(Equation 5) $$VAPE=100\text{\%}\times Var\frac{\left|\hat{Y}-Y\right|}{Y},$$

where M is the number of time steps, N is the number of roads, $\hat{y_{i}^{j}}$, $y_{i}^{j}$ and $\omega_{Ai}^{j}$ respectively represent the prediction result, real value, weight of the *j*th time step in the *i*th road segment, and the vector of $\hat{y_{i}^{j}}$ and $y_{i}^{j}$ is $\hat{Y}$ and *Y*.

#### Square error metrics

Unlike the absolute error metrics, square error metrics use the error square to avoid the complex calculation of absolute values, i.e., mean square error (MSE), root MSE (RMSE), Normalized RMSE, and $R^{2}$.

(Equation 6) $$RMSE=\sqrt{\frac{1}{MN}\sum_{j=1}^{M}\sum_{i=1}^{N}\omega_{Si}^{j}{\left(\hat{y_{i}^{j}}-y_{i}^{j}\right)}^{2}}\text{,}$$

(Equation 7) $$R^{2}=1-\frac{\sum_{j=1}^{M}\sum_{i=1}^{N}{\left(\hat{y_{i}^{j}}-y_{i}^{j}\right)}^{2}}{\sum_{j=1}^{M}\sum_{i=1}^{N}{\left(\bar{Y}-y_{i}^{j}\right)}^{2}}\text{,}$$

where$\omega_{Si}^{j}$ is the weight of the *j*th time step in the *i*th road segment in RMSE, and $\bar{Y}$ represents the average of *Y*. A smaller MAPE or RMSE means better prediction accuracy, and a larger $R^{2}$ represents better prediction effect.

#### Computation time

Based on the training process of deep-learning methods, the computation time can be divided into two parts: inference time and training time. The former refers to the computation time to complete a prediction. The longer the inference time the less valuable it is for online prediction. Besides, the training time reflects the complexity of prediction model, and the light model with accuracy result is the trend of development. Furthermore, since the computation time is related to hardware, it makes sense to compare the computation time under the same hardware conditions.

### Public datasets and open-source codes

Dataset is essential to development and evaluation of prediction models. Table 3 summarizes the public dataset or standard cycle of vehicles applied in different speed predictions. Open-source models can help researchers quickly understand the ideas of existing models and reproduce their methods for comparison. Table 4 provides a list of the public model in different speed predictions to facilitate further research.

**Table 3:** Some public dataset of speed prediction

Dataset	Application	Resolution	Location	Link	Reference
PeMSD4	Traffic	5 min	California, USA	http://pems.dot.ca.gov/	Huang et al., 2020; Yin et al., 2021b; Ge et al., 2019
PeMSD7	Traffic	5 min	California, USA	http://pems.dot.ca.gov/	Yu et al., 2019a; Zhang et al., 2021a; Huang et al., 2020; Ge et al., 2019; Yu et al., 2018
PeMSD8	Traffic	5 min	California, USA	http://pems.dot.ca.gov/	Huang et al., 2020; Yin et al., 2021b
PeMS-BAY	Traffic	5 min	California, USA	http://pems.dot.ca.gov/	Li et al., 2018b; Feng et al., 2020; Chen et al., 2019; Park et al., 2020; Chen et al., 2020a
INRIX	Traffic	5 min	USA	https://inrix.com/	Lin et al., 2018; Cui et al., 2018, 2020
METR-LA	Traffic	5 min	Los Angeles, USA	https://www.metro.net/	Li et al., 2018b; Feng et al., 2020; Chen et al., 2019 Zhang et al., 2018; Park et al., 2020; Pan et al., 2019
DRIVE Net	Traffic	5 min	Seattle, USA	http://uwdrive.net/STARLab	Ma et al., 2018; Qu et al., 2021
Madrid city	Traffic	15 min	Madrid, Spain	https://datos.madrid.es/portal/site/egob/	López Manibardo et al., 2021
GAIA	Traffic	2∼4s	Chengdu & Xian, China	https://outreach.didichuxing.com/research/opendata/en/	Niu et al., 2019
GCM	Traffic	5 min	Gary Chicago Milwaukee, USA	http://www.travelmidwest.com/	Polson and Sokolov,2017
Q-Traffic	Traffic	15 min	Beijing, China	https://github.com/JingqingZ/BaiduTraffic#q-traffic-dataset	Liao et al., 2018
PortoTaxi	Traffic	15 s	Porto, Portugal	https://www.kaggle.com/c/pkdd-15-predict-taxi-servicetrajectory-i	Tang et al., 2020
Seattle Loop	Traffic	5 min	Seattle, USA	https://github.com/zhiyongc/Seattle-Loop-Data	López Manibardo et al., 2021; Cui et al., 2020
NYC	Traffic	5 min	New York, USA	https://www.kaggle.com/crailtap/nyc-real-time-traffic-speed-data-feed	López Manibardo et al., 2021
UDDS	Vehicle	1s	USA	https://www.epa.gov/emission-standards-reference-guide	Liu et al., 2017; Cao et al., 2021; Sun et al., 2017 Liu et al., 2021; Sun et al., 2015a; Yan et al., 2018
OCC	Vehicle	1s	Los Angeles, USA	https://www.arb.ca.gov/regact/bus02/appc.doc	Lian et al., 2017
HWFET	Vehicle	1s	USA	https://www.epa.gov/sites/default/files/2015-10/hwy10hztable.txt	Zhou et al., 2019b; Liu et al., 2021; Sun et al., 2017, 2015a
US06	Vehicle	1s	USA	https://www.epa.gov/sites/default/files/2015-10/us06col.txt	Sun et al., 2017; Liu et al., 2021; Sun et al., 2015a; Yan et al., 2018
JN1015	Vehicle	1s	Japan	https://www.epa.gov/sites/default/files/2015-10/j1015col.txt	Sun et al., 2017
NYCC	Vehicle	1s	New York, USA	https://www.epa.gov/sites/default/files/2015-10/nycccol.txt	Liu et al., 2021
SC03	Vehicle	1s	USA	https://www.epa.gov/sites/default/files/2015-10/sc03col.txt	Liu et al., 2021
NEDC	Vehicle	1s	Europe	https://github.com/dabo248/nedc	Lian et al., 2017; Liu et al., 2021; Sun etal.,2017, 2015a
NGSIM	Vehicle	–	USA	https://data.transportation.gov	Liu et al., 2018b; Zhou et al., 2019a; Shih et al., 2019; Ye et al., 2019

**Table 4:** Some open-source code of speed prediction model

Application	Model	Reference	Year	Framework	Link
Traffic	DCRNN	Li et al., 2018b	2018	Tensorfilow	https://github.com/liyaguang/dcrnn
	GRNN	Wang et al., 2018	2018	–	https://github.com/xxArbiter/grnn
	STGCN	Yu et al., 2018	2018	keras	https://github.com/Knowledge-Precipitation-Tribe/STGCN-keras
	Graph WaveNet	Wu et al., 2019	2019	Torch	https://github.com/nnzhan/Graph-WaveNet
	ST-MetaNet	Pan et al., 2019	2019	MXNet	https://github.com/panzheyi/ST-MetaNet
	T-GCN	Zhao et al., 2020b	2020	Tensorfilow	https://github.com/lehaifeng/T-GCN
	GMAN	Zheng et al., 2020	2020	Tensorfilow	https://github.com/zhengchuanpan/GMAN
Lane-level	MDL	Lu et al., 2020a	2020	–	https://github.com/lwqs93/MDL
	ST-AFN	Shen et al., 2021	2021	–	https://github.com/MCyutou/ST-AFN

### Challenges and future directions

**Figure 6:**
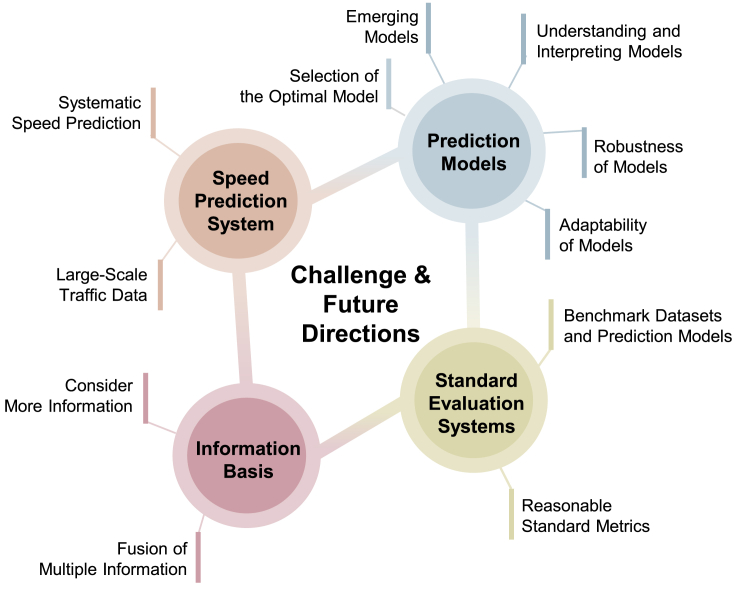
The challenges and future directions of the speed prediction in transportation system

This section points out the existing challenges and future research opportunities of speed prediction in transportation system as shown in Figure 6.

#### Speed-prediction system

##### Systematic speed prediction

Systematic speed prediction aims to combine the speed predictions at different levels, unlike the speed prediction at a single level, to predict the speed at different levels. The different speed predictions can extract the speed patterns at different levels and promote the understanding of dynamic traffic from macro to micro. First, systematic speed prediction can enhance the deterministic part of the ground truth and extend applications with the help of the information at different levels, especially for Intelligent Vehicle Infrastructure Cooperative Systems (Zhang et al., 2021b). Traffic-speed prediction has been combined with vehicle-speed prediction to improve the prediction performance in (Shao and Sun, 2021; Jiang and Fei, 2017), but the further application of systematic speed prediction is still insufficient and encouraged. Second, a comprehensive understanding of traffic can promote further model development and drive them forward. Besides, current systematic exploration can serve as the basis for the speed prediction of future traffic participants, such as free crowd flow, two-wheeled vehicles, intelligent vehicles, and unmanned air vehicles moving in three dimensions.

##### Large-scale traffic data

The various spatio-temporal data in traffic makes systematic speed prediction feasible. However, large-scale data brings enormous challenge on data processing. Thus, edge computing is regarded as the future framework of speed prediction. Edge computing has been applied to predict the urban energy in (Lee et al., 2019; Luo et al., 2019), but it is necessary to continue further research on edge computing in systematic speed prediction. In addition, the traffic data involves personal privacy and national security, and the huge amount of data means huge information-security issues (Zhang et al., 2021a). Moreover, privacy protection implies a reduction of computation efficiency, and it is essential to consider the trade-off between efficiency and privacy security.

#### Information basis

##### Various information

The information and knowledge used for prediction determine the predictable part of the ground truth. The evolution of the prediction model corresponds to the increase of the information used in the model. However, most of the existing studies consider the external factors individually and ignore the coupled relationship between various factors. Thus, it is become important to further extract the complex traffic patterns. Moreover, ITS is a potential way of information collection, which is able to achieve real-time collection across space and enlarge the single-vehicle view. Concerning traffic knowledge, existing methods mostly focus on spatio-temporal dependency. Furthermore, extracting spatio-temporal dependency at a larger scale is still a future direction. The prediction research should be extended from short-term to long-term in the temporal dimension, and from a single vehicle, lane, road segment to the road network in the spatial dimension. Moreover, the joint spatio-temporal dependency is urgently needed in future research. Furthermore, in addition to spatio-temporal dependency, more systematic features of influencing factors should be considered.

##### Fusion of multiple information

The complementarity of multi-sources information can compensate for missing or noisy data. With regard to the fusion method, feature-level fusion can achieve a better performance than data-level fusion (Li et al., 2019a). However, the characteristics (e.g., type, resolution) of multi-sources data are different, and the process methods of different data are the first problem of information fusion. Furthermore, as the information dimensions increase, the difficulty of feature extraction and computational consumption will rise significantly. Therefore, the balance between data requirement and computational efficiency is an important direction.

#### Prediction models

##### Understanding and interpreting models

Nowadays, the mainstream of speed prediction is deep-learning, but it has been criticized for its black-box characteristics. The end-to-end characteristics allow us to reach accuracy results without understanding how to obtain them, which greatly reduces the reliability and transferability of such methods. In practice, one might even prefer the more interpretable but less performant solution (López Manibardo et al., 2021). A potential direction is to map the physical features of information and knowledge into the deep-learning network or input. GCN replaces the input from the image to the graph based on non-Euclidean features of the road network and improves the prediction accuracy. Reference (Wang et al., 2019; Kim et al., 2019; Lee et al., 2020) took advantage of the road network topology to build up the deep-learning network. However, prediction models with good interpretability need to be further explored.

##### Robustness of models

The robustness of prediction models usually refers to the ability to resist perturbations and noise. During the data collection, data is inevitably missing and noisy due to environmental influences and sensor limitations. Most of the studies separated the data processing (i.e. missing and noise handling) from the model itself (Wang and Shi, 2013; Zhao et al., 2019a; Chen et al., 2020b), and some work treated this part as a new learning task (Tian et al., 2018; Zhang et al., 2021d). Besides, it is a future direction for the development of model robustness to combine data imputation with prediction methods (Zhang et al., 2021d; Chen and Sun, 2021; Boquet et al., 2020). However, data imperfections are inevitable in realistic environments, thus developing predictive models with good robustness requires more effort.

##### Adaptability of models

The adaptability describes the working range of the scenarios prediction methods covered. The first is the adaptability to the different structures of road networks. For example, the traffic patterns on urban roads are more complex than that of freeways due to the complex intersection and traffic light control (Jin, 2010), leading to their difference of traffic pattern. The second is the adaptability to different traffic scenarios, especially the rare scenarios, such as the accident scenario. The adaptability to different vehicles (e.g. emergency vehicles (Zhao et al., 2020a) and trucks (Zhao et al., 2019b)) is also significant. In addition, the adaptability to the change of traffic patterns is also worth noticing. Our city is developing all the time, and the reshaping of road leads to the old traffic data being useless, which is defined as concept drift (Lana et al., 2018). Therefore, prediction models should adapt to the new traffic data, and some graph-based prediction methods adopted dynamic input matrix to model the dynamic spatio-temporal dependency (Diao et al., 2019; Zheng et al., 2020). It is necessary to continue further research in this direction. Finally, as data-driven methods do not require explicit modeling, the speed prediction models can also be applied to other prediction and promote the development of prediction method.

##### Emerging models

A number of deep-learning methods have been adopted for speed prediction, such as Transformers, Generative Adversarial Networks (GAN), Meta learning. Transformers (Xu et al., 2021) is based on Encoder-Decoder structure and can achieve parallel computation compared to RNN thanks to the self-attention mechanism. GAN (Yu and Gu, 2019; Zhang et al., 2021c) captures the data distribution by two networks competing with each other and exhibit strong robustness. Meta learning (Pan et al., 2019) possesses self-learning capability to leverage experience to guide future tasks. Even though most of them in speed prediction are still in their infancy, those methods inject new life into speed prediction.

##### Selection of the optimal model

How to choose the right method for a certain problem needs further exploration. No one model is suitable for any problem, and a method is a suitable choice only if it reaches the trade-off between the benefits and cost (López Manibardo et al., 2021). This article attempts to present the existing methods in terms of the utilization of different information, but the real problems require additional considerations such as hardware limitations, real-time performance, data needs, accuracy, etc. How to balance these requirements to select the right model requires further research. Meanwhile, most of the existing work has focused on the design of new models, but not enough attention has been paid to the model parameter optimization. The potential of existing models needs to be further explored. In addition, the trend of existing models is toward more complex. Most works mechanically stack spatio-temporal layers to extract spatio-temporal dependency. However, considering the practical applications and real-time requirements, light prediction models are also an important direction.

#### Standard evaluation systems

Many exciting methods have emerged from the research wave of the data-driven method, but the development of speed prediction is based on the side-by-side comparison of different methods. However, the existing methods have different experimental datasets and evaluation metrics, which makes it difficult to state whether the performance improvement is thanks to parameter tuning or model improvement. Therefore, a benchmark evaluation system is urgently needed, including standard datasets, prediction models, and reasonable evaluation metrics.

##### Benchmark datasets and prediction models

Benchmark datasets can standardize the experiment and compare the performance of individual methods in the same arena. In addition, benchmark-dataset management can directly address the issues mentioned in Section 6.3.2 by itself, such as the lack of external factors, the inaccuracy of existing datasets, etc. The standard prediction model delineates an evaluation reference for the benchmark evaluation, and reduces the workload of comparison experiments.

##### Reasonable standard metrics

A reasonable evaluation system is essential for identifying model problems and guiding model development. However, the current evaluation mostly focuses on accuracy evaluation. In fact, the various performances of practical application should be evaluated. In addition, the evaluation metrics should not be limited to the result evaluation but can further evaluate the performance of internal system characteristics. For example, reference (Wu et al., 2018) proposed a metric to measure the prediction performance of spatial and temporal distribution. Moreover, the model’|'s potential should be fully explored to provide other valuable information and enhance the result reliability.

## Conclusion

This study conducted a comprehensive review of speed prediction at different levels in the transportation system. Specifically, this article analyzed the information used and the prediction model on the prediction results. First, the speed at different levels i.e. traffic (macro), vehicle (micro), and lane-level (meso) aims to describe the dynamic traffic, but from different perspectives. Therefore, the main focus of prediction methods is from the spatio-temporal dependency and external factors modeling to temporal dependency and internal factors modeling, especially to the that of micro driving behavior. In addition, the article summarized the prediction methods at different levels based on how prediction models use the different information to meet the challenge of different prediction problems. Furthermore, the article reviewed the existing evaluation metrics, public datasets, and open-source prediction models. Finally, the existing challenges and future directions of speed prediction were discussed in detail. To the best of our knowledge, this article is the first review for speed prediction at different levels, and the lane-level speed prediction is the first time to be elaborated in a review. Therefore, this article can help researchers quickly locate their research in this field and find potential branches to further explore. We hope that the discussion of speed prediction with a systematic perspective can probe the pulse of traffic and improve city transportation.

### Limitations of the study

This article focuses on the systematic review of speed prediction methods at different levels in transportation system. A quantitative comparison of different prediction method in specific scenarios are beyond the reach of this article and require further research.
